# Injury has a lower incidence but higher burden than illness in elite South African netball players: A prospective cohort study

**DOI:** 10.17159/2078-516X/2025/v37i1a20189

**Published:** 2025-05-15

**Authors:** DH Pistorius, A Jansen van Rensburg, DC Janse van Rensburg, G Ramkilawon, D Ramagole

**Affiliations:** 1 Section Sports Medicine, Faculty of Health Sciences, University of Pretoria, South Africa; 2 Medical Advisory Panel, World Netball, Manchester, United Kingdom; 3 Department of Statistics, Faculty of Natural and Agricultural Sciences, University of Pretoria, South Africa

**Keywords:** female sport, epidemiology, team, professional sport

## Abstract

**Background:**

Netball poses significant injury risks with explosive movements and restricted court areas. Team sports with frequent contact predispose players to illness.

**Objectives:**

Over six months, the period prevalence, incidence, clinical characteristics, and severity of injuries and illnesses in elite South African (SA) netball players were investigated.

**Methods:**

A six-month prospective cohort study followed 24 elite female national team players (age 25.7±4.8 years). Injury and illness data were self-reported via a two-weekly online survey. Main outcome variables included period prevalence (PP, %), incidence (I; injury/1000 player-hours; illness/1000 player-days), severity (time-loss in days), injury burden (InjB; days lost/1000 player exposure-hours) and illness burden (IllB; days lost/1000 player exposure-days).

**Results:**

In total, 26 injuries were reported (PP=9%; I=5.4). Lower limb injuries (83%), specifically the knee (44%), involving joint sprains/ligament tears (28%), mainly occurred. Most injuries (55%) were sustained during match play (56%) and due to player contact (39%). Goal defenders suffered most injuries (22%). Injuries resulted in mild time-loss (1–7 days), with an InjB of 3.5 days lost/1000 player exposure-hours. In total 33 illnesses were reported (PP=11%, I=6.9), mainly involving the upper respiratory tract (85%). Illness severity was mostly minimal, with no time-loss (52%), resulting in an IllB of 17.3 days lost/1000 player exposure-days.

**Conclusion:**

This study provides important descriptive injury and illness information in elite netball players. InjB translates to 14.9 days lost, and IllB to 3.1 days lost during the six months. Player contact is a major cause of injury. Lower limb injuries, contact prevention and upper respiratory tract illnesses should receive attention in preventative strategies. More injury and illness data in elite netball is needed.

Netball is a physically demanding team sport characterised by sudden acceleration, deceleration, jumps and turns, with a high injury risk.^[2]^ Despite netball not being categorised as a contact game, competing for the ball leads to frequent contact-related medical encounters.^[2]^ It is a popular sport, mainly amongst females in Commonwealth countries. ^[3]^

Various Injury and illness surveillance is an essential aspect of sports medicine. It forms part of the concerted efforts of the International Olympic Committee (IOC) to protect the athlete’s health.^[4]^ Surveillance plays a pivotal role in prevention by identifying the nature of the injuries and illness and the setting in which they occur, and, ultimately, is the first step in individualising risk management in sport.^[4]^

Despite its popularity and the high risk of injury and illness, there are few netball surveillance studies compared to sports like rugby, soccer and basketball. Most injury surveillance studies focus mainly on the competition setting without exploring training aspects.^[2,5–6]^ Previous studies have shown that most injuries occur during match play, with the ankle being the most commonly injured anatomical structure, followed by the knee.^[1–3,6–9]^ Contact with another player is a common mechanism of injury (MOI).^[2–3,5,9]^ Other mechanisms include inappropriate landings, falls and ball contact.^[3,9]^ Patterns in the MOI can guide us in training, skills development and injury prevention.^[9]^

Netball players mostly share facilities, which may increase infection risk. Illness may lead to time loss in training, poor performance, increased risk of injury, and added psychological stress, potentially placing a notable burden on teams to stay competitive.^[10]^ In elite sports, the most common system affected is the respiratory tract, followed by the gastrointestinal tract. ^[2,10]^ A study of illness risk factors in athletes preparing for the summer Olympics across multiple categories identified females as having an increased risk of illness compared to male athletes.^[10]^

There is a notable gap in the literature regarding studies prospectively reporting injuries and illness in netball players during training and match preparation for an elite tournament. This study investigates the prevalence, incidence, clinical characteristics, and severity of injuries and illnesses in a cohort of elite South African netball players over six months in preparation for the 2023 Netball World Cup.

## Methods

### Study design and ethical concerns

Data was collected from elite South African netball players in a prospective observational cohort study. All participants received information about the study before data collection began and gave consent for their data to be used for research.

Netball South Africa approved the conduct of the study, and the Research Ethics Committee of the University of Pretoria approved the research (REC 466/2019 and REC 313/2022). Fear of being excluded from the team when self-reporting injury and illness data raises an ethical concern. It was emphasised that data collection for the study will not affect team selection and will only be verified with the medical team when there are health implications for the player. No incentives were offered for participation.

### Participants

The cohort included 24 elite female netball players selected for the South African national team. The selected team players did not stay together but followed their regional netball schedule and joined scheduled training camps in different parts of South Africa.

### Data collection

Baseline demographic information collected at the start of the study (Supplementary A) included age (years), weight (kg), height (m), BMI (kg/m^2^), previous injuries and illness, chronic illness, and previous COVID (in the six months before the study).

The injury and illness survey (Supplementary B) was compiled using Qualtrics software.^[11]^ A link to the survey was shared by team management to the team’s communication group. All participants self-reported their injuries and illnesses every 14 days via the online survey, independent of attending training camps and matches in the previous two weeks. When low response rates were noted, reminders to complete the online survey were distributed to players via the team’s communication group. No incentives were offered. Data were collected over six months, from May to November 2022, in preparation for the 2023 Netball World Cup. The International Olympic Committee consensus statement and the OSTRC-H questionnaire guidelines for reporting epidemiological data were followed.^[4,12]^

Injury data recorded included anatomical region, body area, pathology type, onset, severity (number of days lost and perceived severity), setting where the injury occurred, MOI, court area and player position at the time of injury. Illness data included the organ system involved and the severity of illness (number of days lost and perceived severity). We also recorded match play and training time in hours. Not all OSTRC-H questions were included to minimise the questionnaire length and prevent loss of interest. However, this action prevented calculating a perceived severity score in the results.

### Definitions

This study’s definitions of injury and illness align with the 2020 IOC consensus statement and the OSTRC-H questionnaire:^[4,14]^

*Injury:* “Tissue damage or other derangement of normal physical function due to participation in sports, resulting from rapid or repetitive transfer of kinetic energy.”^[4]^
*Illness:* “A complaint or disorder experienced by an athlete, unrelated to injury.”^[4]^
*Time-loss: “An* injury or illness resulting in ≥1-day loss of training or competition.”^[4]^
*Severity:* “Number of days that the athlete was unavailable for training and competition, from onset until the athlete was fully available for training and competition.” Time loss was grouped in time clusters of 0 days (minimal), 1–7 days (mild), 8–28 days (moderate) and >28 days (severe).^[4]^
*Perceived severity:* Players’ perception of the severity of an injury was appraised as ‘full participation without problems’, ‘full participation with problems’, ‘reduced participation’, and ‘could not participate at all’.^[12]^ A gatekeeping function was added – if a player selected “full participation without problems”, no further injury data were collected. No gatekeeping was implemented for illness.
*Injury burden (InjB):* Total number of days lost due to injury/1000 player exposure hours.^[4]^
*Illness burden (IllB):* Total number of days lost due to illness/1000 player exposure days.^[4]^

### Calculation of player exposure

*Training hours:* The average training hours were calculated from the number of sessions each player reported in the survey. If players missed a training camp and reported fewer sessions, the average hours of the entire squad decreased.

*Match hours:* Self-reported match play is the average of each player in the entire squad’s number of matches played. There are 24 players in the squad and only seven on the court during a match, with limited substitutions allowed per match. Therefore, the average number of matches played per player will be lower than the total number of matches played by the team.

*Player exposure:* the total exposure for both injury and illness was assessed over a 6-month period, and calculated as follows:

- Injury exposure: The total exposure hours were calculated based on each player’s participation in matches and training sessions, expressed per 1000 player-hours (95% CI). This was determined using the formula: ^[4,13]^
Total injury exposure hours=(number of matches+number of training sessions)×session duration in hours
  For consistency, each match was considered equivalent to one hour of play. Since individual participation varied depending on training camp attendance and match selection, exposure hours were calculated separately for each of the 24 players and then summed to determine the total player-hours for the full squad.
- Illness exposure: The total illness exposure days were calculated as the total number of days in the 6-month assessment period multiplied by the 24 squad members, expressed per 1000 player-days (95% CI). This was determined using the formula: ^[4]^
Total illness exposure days=Assessment period in days×24.

### Main outcome variables reported

We report the response rate (%) for the two-weekly survey period; the netball-related injury and illness frequency (n=%); period prevalence (% players; 95% CI); incidence (injury/1000 player-hours; 95% CI; illness/1000 player-days; 95% CI); severity; InjB, and IllB.

### Statistical data analysis

The statistical program R (version 4.1.3 released 2022; http://www.r-project.org/ for Mac) was used for data analysis. Descriptive statistics such as the mean, standard deviations, frequencies and proportions were used. Specific focus was placed on the types of injuries and illnesses and their respective incidence and prevalence.

The incidence of injuries is expressed as the number of injuries/1000 player-hours (95% CI), and the incidence of illnessas the number of illnesses/1000 player-days (95% CI). InjB was reported as the number of days lost/1000 player exposure hours (95% CI), and IllB as the number of days lost/1000 player exposure days (95% CI).

## Results

### Response rate

The survey was distributed 15 times over six months, yielding 287 responses out of an expected 360, resulting in a mean participant response rate of 80%.

### Demographics

Table 1 depicts the baseline demographics of the participants. Eight previous injuries, nine previous illnesses, one chronic disease, and one residual COVID-symptom case were reported. None of these were included in the subsequent data collection, and no recurrences occurred during the study period

### Injury and illness exposure

The mean training time per 2-week cycle was 14.6±10.5 hours with a mean match time of 2.2±1.8 hours, equating to a mean total netball exposure of 16.7±10.9 hours/2-week cycle. The study period lasted 198 days. The total injury exposure was 33 621 player hours, and illness exposure was 4752 player days for the 24 participants over the six months.

### Injury epidemiology

Over the six months, 24 players reported 26 injuries. Six reported the perceived severity as “full participation without problems”, and two injuries had incomplete data and were therefore excluded from further injury data analysis. The injury incidence was 5.4/1000 player hours (95% CI: 3.5–7.7) with a mean injury period prevalence of 8.9% (95% CI: 3.9–13.8).

### Clinical characteristics

Table 2 presents the clinical characteristics of the injuries. The main anatomical region injured was the lower limb (83%), with the knee (44%) and ankle (33%) the two most commonly injured body areas. Ligament/joint capsule and muscle/tendon were the most common tissue types injured (28% each). Joint sprains/ligament tears (28%) and muscle injuries (22%) were the most common pathology types. Injury onset was mainly sudden (78%).

### Severity of injuries

The players mainly reported perceived injury severity as “*full participation with problems*” (35%). Looking at time loss, injury severity was mostly mild (55%). The only severe injury was knee-related (Table 3). The overall InjB was 3.5 days lost/1000 player exposure hours (95% CI: 2.9–4.1).

### Injury setting, action and positions played during injury

Injuries mainly were sustained during match play (56%) and following contact with another player (39%), either from the same (22%) or the opposing team (17%). Goal defence sustained the most injuries (22%), followed by centre (17%)and goal shooter (17%). The combined position groups (defenders, centre court and shooters) sustained similar amounts of injuries. (Table 4)

### Illness epidemiology

Over the six months, 24 players reported 33 illnesses. In some instances, multiple system involvement occurred (Table 5). The illness incidence was 6.9/1000 player-days (95%CI: 4.8–9.26) with a mean illness period prevalence of 11% (95% CI: 6.3–15.5).

### Clinical characteristics

Players mainly reported illnesses in the upper respiratory tract (56%), followed by the lower respiratory tract (18%) and gastrointestinal system (10%) (Table 5). Due to the low number of injuries and illnesses during the study period, risk factor analysis was not possible.

### Severity of illness

Despite illness symptoms, the players mainly rated perceived severity as “full participation without problems” (33%). More than half of the reported illnesses were minimal, with no time loss, followed by mild severity (42%), which resulted in 1–7 days of time loss from training and competition (Table 6). The overall IllB was 17.3 days lost/1000 player exposure days (95% CI: 13.7–21.0).

## Discussion

This study aimed to prospectively investigate the period prevalence, incidence, clinical characteristics, and severity of injuries and illnesses in 24 elite netball players over six months. The main findings included a period prevalence of 9% for injury and 11% for illness. Almost half of the reported injuries (56%) and illnesses (42%) caused training and match play time loss of 1–7 days. The InjB was 3.5 days lost/1000 player exposure hours, and IllB was 17.3 days lost/1000 player exposure days. This translates to 14.9 days lost due to injury and 3.1 days lost due to illness during the six months.

The present study on a cohort of 24 female players (mean age of 25.7 years) reported an injury incidence of 5.4/1000 player-hours, which is comparable to another Commonwealth-based study on Australian netball premier club players (mean age of 25 years), who reported an injury incidence of 5.2/1000 player-hours over one season.^[9]^ A South African study of players ranging from under-18 (U18) to senior players reported an overall injury incidence of 33.9/1000 player-hours over two consecutive seasons.^[8]^ The results of the present study are lower than those of a study on elite players during the 2019 Vitality Netball World Cup tournament, reporting an injury incidence of 54.8/1000 player-hours.^[2]^ The difference in injury incidence may be due to elite vs amateur level of play, a difference in consensus on defining injury, different data collection methods, or different data collection settings. Tournaments may also have a higher injury rate than standard match play due to consecutive matches without adequate recovery time. ^[5]^

The most frequently injured anatomical region was the lower limb (83%), specifically the knee (44%) and ankle (33%). These are consistent findings throughout injury surveillance studies in netball; however, most studies report higher ankle than knee involvement.^[1–2,5–6,8–9,15–16]^

Knee injuries caused the most time-loss, which correlates with an Australian study of injuries sustained in professional netball players over three seasons.^[15]^ The nature of the sport, with explosive movements, abrupt stops, frequent contact in a confined court space, and strict footwork rules, predispose the lower limb to injury. This highlights the importance of proprioception and lower limb strength training in injury prevention.^[17]^

More than half of all injuries were mild, resulting in 1–7 days lost (56%). Translating the player’s perceived severity, the impact of most injuries resulted in full participation with problems (35%). The severity in this study differs from a South African study on sub-elite players over two consecutive seasons that reported most injuries (40%) as moderate, leading to 8–28 days of time loss.^[8]^ Conversely, in elite players, most injuries (67%) at the 2019 Vitality Netball World Cup did not lead to a time loss.^[2]^ The variation in injury severity across studies may indicate a need for standardisation in injury data collection. The overall InjB in the present study was 3.5 days lost/1000 player exposure hours, which translates to approximately 14.9 days lost due to injury in the six months.

Consistent with previous Commonwealth-based studies, most injuries (56%) occurred during match play.^[8–9,16]^ Although netball is classified as a non-contact sport, most injuries were related to contact with another player (39%) from the same team (22%) or the opposite team (17%). This finding concurs with previous studies on elite and sub-elite players.^[2,5,8]^ Fast movements while competing for the ball in a restricted area led to unintentional player contact, even with rules to penalise contact.

The goal defence (22%), centre and goal shooter positions (17% each) suffered the most injuries. Previous studies reported the centre as the most injured position.^[2,6,8]^

The present study reports an illness incidence of 6.9/1000 player-days. This study results are comparable to elite players in the 2019 Vitality Netball World Cup tournament, with an illness incidence of 7.6/1000 player days.^[2]^ Another four-day high school netball tournament study reported an illness incidence of 0.55/1000 player-days.^[18]^ Upper respiratory tract illnesses (URTI) were mostly reported (56%). Although most illnesses during the 2019 Vitality Netball World Cup tournament were non-respiratory (64%), upper respiratory tract infection was still the most common specific diagnosis (36%).^[2]^ The most frequent body systems reported at a four-day high school netball tournament were digestive (39%) and respiratory (15%).^[18]^ The present study’s results align with several studies across sporting codes that report athletes are mostly affected by respiratory system illness.^[10,19–20]^ Despite the strong netball culture of Commonwealth countries, no other Commonwealth-based studies on illness in netball were found.

Most illnesses (64%) were mild, resulting in 1–7 days of time loss. The high school and 2019 Vitality tournaments mostly reported no time loss due to illness.^[2,18]^ Based on the players’ perceived severity, they mainly reported full participation despite illness symptoms (61%). The overall IllB in this study was 17.3 days lost/1000 player exposure days, approximately 3.1 days lost due to illness in the six months. No other netball studies reporting IllB were found. Time loss due to illness has detrimental performance and psychological consequences, emphasising the importance of URTI prevention measures. Especially in team sports, close contact on and off the court is inevitable.

### Study strengths and limitations

The main strength of this study is the prospective cohort design over six months, collecting injury and illness data in elite netball players, both in and out of competition. These results used the reporting methods published in the IOC consensus statement,^[4]^ enabling data comparison across studies. The present study is the only study reporting players’ perceived severity according to modified OSTRC-H questions^[12]^ and not only time loss. The self-reporting data collection enabled us to collect data from a cohort of players over a prolonged period despite diverse geographical locations. Reliance on self-reported data introduces possible bias, including potential underreporting or inaccuracies due to subjective injury/illness severity interpretation, errors in question interpretation, loss of interest in the study, fear of being left out of the team and different subjective perceptions of what an injury or illness entails. It could further contribute to the relatively low injury and illnessincidence reported throughout the six months. The average training hours and number of matches played per 14-day cycle do not reflect the team’s total training hours and matches. The squad consisted of 24 players, but only seven were on the court during a match (excluding substitutions). The players who missed training camps or did not play all the matches influenced the average training hours and matches played by the entire squad. This implicates a variability in reported exposure when calculating player exposure hours and may skew injury incidence rates. The small cohort of 24 players also restricts the generalisability and statistical impact. The low injury and illness numbers reported preclude risk factor analysis. Not all OSTRC-H questions were included in the survey, which prevents the calculation of a perceived severity score and comparison with other self-reporting research studies following the OSTRC-H guidelines.^[12]^ Illness burden is a compounded value, looking at overall severity and general player-exposure days, without accounting for seasonal trends in certain diseases’ prevalence and differentiating between different disease severities.

## Conclusion

This six-month prospective study provides important descriptive information regarding the epidemiology of injuries and illnesses in elite netball players. Netball players are mainly affected by lower limb injuries and upper respiratory system illnesses. The dominance of lower limb injuries emphasises the need for lower limb injury prevention programs. Time loss due to a specific injury or illness mostly accounts for 1–7 days. Also, injury had a lower incidence but a higher burden than illness and contact with another player remains the most common cause of injury. More injury and illness data are needed to substantiate the conclusions. More injury and illness data are needed in elite netball, specifically during training and match preparation, to enhance future risk factor analysis. Risk factor analysis will aid in developing effective injury and illness prevention measures.

### Practical implications

- Player contact is a major cause of injury. Players should be trained to avoid unintentional contact on the court.
- Injury prevention programs should focus on the lower limb.
- Upper respiratory tract illness prevention measures are important, especially in team sports.

## Supplementary Information





## Figures and Tables

**Table 1:** Baseline demographic data of all female netball participants (n=24)

Characteristics		All participants

Age (years), mean (±SD)		25.7±4.8
Weight (kg), mean (±SD)		71.6±7.3
Height (m), mean (±SD)		1.8±0.1
BMI (kg/m^2^), mean (±SD)		22.5±1.4

Previous injuries (in 6 months prior to study), n (%)	No	16 (67%)
	Yes, upper limb	2 (8.3%)
	Yes, lower limb	6 (25%)

Previous illness (in 2 months prior to study), n (%)	No	15 (63%)
	Yes	9 (38%)

Chronic illness, n (%)	No	23 (96%)
	Yes	1 (4.2%)

COVID (in 6 months prior to study), n (%)	No	14 (61%)
	Yes, with residual symptoms	1 (11%)
	Yes, without residual symptoms	8 (89%)

n, number; SD, standard deviation; BMI, body mass index. Chronic illness: A disease or condition of long duration and generally slow progression that requires ongoing medical attention.^[14]^

**Table 2:** Clinical characteristics of injuries in elite female netball participants

Region	Body area*	Number of injuries n=18 (%)

Head and neck	Neck	1 (5.6%)

Upper limb	Hand/fingers	2 (11%)

Lower limb	All	15 (83%)
	Knee	8 (44%)
	Lower leg	1 (5.6%)
	Ankle	6 (33%)

**Tissue**	**Pathology Type** *	**Number of injuries n=18 (%)**

Cartilage/Synovium/Bursa	Cartilage	2 (11%)

Bone	All	2 (11%)
	Fracture	1 (5.6%)
	Stress fracture	1 (5.6%)

Ligament/Joint capsule	Joint sprain/ligament tear	5 (28%)

Muscle/Tendon	All	5 (28%)
	Muscle contusion	1 (5.6%)
	Muscle injury	4 (22%)

Non-specific	Other	4 (22%)

**Injury onset** *	**Type**	**Number of injuries n=18 (%)**

Injury onset	Gradual	4 (22%)
	Sudden	14 (78%)

* 2 missing values. n, number of injuries reported; %, injury frequency (%) reported in the study

**Table 3:** Severity of injury measured in perceived severity and time-loss

Severity	Number of Injuries, n (%)	Anatomical region (n)

**Perceived severity (n=26)**		
Full participation without problems	6 (23%)#	—
Full participation with problems	9 (35%)	—
Reduced participation	5 (19%)	—
Could not participate at all	6 (23%)	—

**Time-loss (≥1-days missed) (n=18)** *		
0 days (minimal)	3 (17%)	Hand/fingers (1), Ankle (2)
1–7 days (mild)	10 (56%)	Neck (1), Hand/fingers (1), Knee (5), Ankle (3)
8–28 days (moderate)	4 (22%)	Knee (2), Lower leg (1), Ankle (1)
>28 days (severe)	1 (5.6%)	Knee (1)

* 2 missing values in total. N, number of injuries reported; %, injury frequency (%) reported in the study.

# 6 injuries perceived as "full participation without problems" were excluded from further injury data collection through the gatekeeping function.

**Table 4:** Injury setting, action and positions played during injury

Setting when injury occurred*		Number of injuries n=18 (%)

Match play		10 (56%)

Non-netball related exercise		3 (17%)

Training		5 (28%)

**Mechanism of Injury (MOI)** *		**Number of injuries n=18 (%)**

Contact with another player	All	7 (39%)
	
	From opposing team	3 (17%)
	
	From same team	4 (22%)

Contact with the ball		1 (5.6%)

Jumping/Landing		4 (22%)

Sudden change in direction		3 (17%)

Other		3 (17%)

**Court Area**	**Player Position** *	**Number of injuries n=18 (%)**

Defenders	Goal Defence	4 (22%)

	Goalkeeper	1 (5.6%)

Centre Court	Wing Defence	0 (0%)

	Centre	3 (17%)
	
	Wing Attack	1 (5.6%)

Shooters	Goal Attack	2 (11%)

	Goal Shooter	3 (17%)

Not related to player position		4 (22%)

* 2 missing values

**Table 5:** Systems involved in illnesses

Systems		Number of symptoms reported per system n (%) *
**All Systems involved**		**50 (100%)**
Neurological		3 (6.0%)
Psychological		1 (2.0%)
Respiratory tract	All	37 (74%)
	Upper	28 (56%)
	Lower	9 (18%)
Cardiovascular		1 (2.0%)
Gastrointestinal		5 (10%)
Musculoskeletal (Not injury related)		2 (4.0%)
Non-specific		1 (2.0%)

* Players could report more than one system involvement per illness

**Table 6:** Severity of illness measured in perceived severity and time-loss

Severity	Number of illnesses n (%)

**Perceived severity (n=33)**	

Full participation without problems	11 (33%)
Full participation with problems	9 (27%)
Reduced participation	9 (27%)
Could not participate at all	4 (12%)

**Time-loss (≥1-days missed) (n=33)**	

0 days (minimal)	17 (52%)
1–7 days (mild)	14 (42%)
8–28 days (moderate)	1 (3%)
>28 days (severe)	1 (3%)
