# Supplementary material for: Injury has a lower incidence but higher burden than illness in elite South African netball players: A prospective cohort study
Source: S Afr J Sports Med. 2025 May 15;37(1):v37i1a20189. doi: 10.17159/2078-516X/2025/v37i1a20189 (PMC12077821; doi:10.17159/2078-516X/2025/v37i1a20189)
Supplement: Supplementary file 1 [file 2078-516x-37-v37i1a20189-s001.pdf]

# Injury has a lower incidence but higher burden than illness in elite South African netball players: A prospective cohort study

This form will provide you with more information on the project and ask your consent to participate. You will only have to complete this form once. Thereafter you will receive a separate questionnaire every 2 weeks. All questionnaires will only take 10 minutes to complete. Thank you for taking your time to read through the information and to answer the questions below.

**What is the project about?**
This project will monitor injuries and illnesses in the team over a period of 6 months whilst the team is preparing for participation in the upcoming international games. We will use an electronic survey to collect information on your health. You will be asked to complete the survey every two weeks. The project aims to identify common injuries and illnesses that affect the team member's ability to perform optimally. By identifying these factors, we can get a better understanding of how to prevent them, helping us to protect your health.

**Who is undertaking the project?**
The project is conducted by Dr Danielle Pistorius and Dr D A Ramagole at the University of Pretoria, Section Sport Medicine, Faculty of Health Sciences. Dr Pistorius is currently doing a Masters degree in Sports Medicine (MSc) and Dr Ramagole is a lecturer in the department and will be supervising the research project.

**Who will take part in the project?**
South African based National Netball team players for the year 2022.

**What will I be asked to do?**
You will be asked to complete an online survey, that will be emailed to you every 2 weeks. The survey will take approximately 5 minutes to complete. It will consist of structured questions where you can tick 'yes' or 'no', and a dropdown menu, where applicable responses to identify a problem will be listed. You will not have to type long responses. The questions are structured to identify the type of injury or health concern and the effect it has on your ability to participate in training.

**Are there any risks associated with participating in this project?**
There are no foreseeable risks identified. Your data will be reported anonymously. With your permission, your health and injury concerns may be shared with the medical personnel associated with the team, to ensure the necessary optimal treatment.

**What are the benefits of this research project?**
By proactively identifying health concerns like injuries and illness in the team, we can address them early and identify the risk factors that cause them. By doing this we can prevent serious illness and injury and help optimise your ability to perform to your potential as an athlete.

**Can I withdraw from the study?**
Participation is voluntary, you are not obliged to participate. You can withdraw from the study without any detrimental consequences to yourself. We do however ask that you strongly consider participating, as your participation will help us monitor the overall health of the team, which in turn will assist with preventing injuries and illnesses where possible.

**What about my personal information?**
Dr Pistorius will request your contact information to share the electronic survey and to contact you in case of any health concerns. No personal information will be shared with external third parties. Your health information may be shared with your team's medical personnel to assist you with any concerns. Overall findings and publication of the findings will be reported anonymously.

**Who do I contact if I have any questions?**
Dr Danielle Pistorius:

Phone: 0828671388

Email: danpistorius@gmail.com

# Informed consent

STUDY TITLE: A PROSPECTIVE COHORT STUDY ON INJURY SURVEILLANCE IN THE SOUTH AFRICAN NATIONAL NETBALL TEAM

I hereby confirm that I have received, read and understood the above written information (Participant's Information) regarding the research study. I am aware that the results of this study, including personal details, will be anonymously processed into a study report. I may, at any stage, without prejudice, withdraw my consent and participation in the study. I have the opportunity to ask questions and am prepared to participate in this study.

**Enquiries:**

Dr Danielle Pistorius

Contact number: 0828671388

Email address: danpistorius@gmail.com

**Participant data**

The information below will be collected only at the start of the study. The information will not be shared with any third party. (List of information collected is condensed below).

1. ID Number, Age, Height in cm, Current weight in kg, At what age did you start playing Netball?
2. Indicate the positions you usually play?
3. Did you sustain any injuries in the past 6 months?
4. Please specify Upper Limb or Lower Limb
5. Do you have any CURRENT injuries? (An injury can be seen as any physical or structural damage to your body, or physical complaint, affecting your ability to participate in sport.)
6. If yes, please specify the diagnosis of the injury, or main symptoms if no diagnosis was made
7. Please specify the LOCATION of the injury, for example "knee" and SIDE if applicable
8. Have you had any operations? Please specify
9. Do you have any allergies? Please specify
10. Were you ill in the past 2 months?, If yes, what illness did you have? *Check all that apply.*
    (Upper respiratory condition like Flu; Gastrointestinal like Diarrhoea; Urinary tract; Gynaecological; Lower respiratory tract like pneumonia; Other)
11. Do you suffer from any chronic medical conditions? (Example: hypertension, asthma, depression) If yes, please specify.
12. Please list all the medication and supplements you currently use?
13. Do you use any contraceptive therapy? If yes, please specify
14. Do you experience regular monthly menstruation?
15. How many menstrual periods did you have in the past 12 months?
16. At what age did you start menstruating?
17. When was your most recent menstrual period?
18. Have you had a stress fracture in the past?
19. Have you ever had problems with your bones, such as low bone density, known as osteopenia or osteoporosis?
20. Have you tested positive for COVID-19 in the past 6 months?
21. If you have tested positive for COVID before, in what month was your most recent positive COVID-19 test?
22. If you tested positive before, do you still experience COVID-19 related symptoms since you tested positive
23. Please indicate which symptoms you still experience? You can choose more than one. (Check all that apply: Loss or change in smell, Loss or change in taste, Coughing, Difficulty in breathing or shortness of breath, Chest pain/pressure, Chest tightness or wheezing, Headaches, Reduced energy/tiredness/fatigue, General muscle pains, Loss of appetite, Loss of concentration or memory, No symptoms, I recovered completely.)
