# Supplementary material for: Injury has a lower incidence but higher burden than illness in elite South African netball players: A prospective cohort study
Source: S Afr J Sports Med. 2025 May 15;37(1):v37i1a20189. doi: 10.17159/2078-516X/2025/v37i1a20189 (PMC12077821; doi:10.17159/2078-516X/2025/v37i1a20189)
Supplement: Supplementary file 2 [file 2078-516x-37-v37i1a20189-s002.pdf]

Injury has a lower incidence but higher burden than illness in elite South African netball players: A prospective cohort study

South African National Netball Team Health Questionnaire

Thank you for taking part in the 2 weekly questionnaires we are sending. We appreciate your contribution to injury and illness surveillance in female netball players. Kindly take time to complete this questionnaire as thoroughly as possible. If you had more than one injury or illness in the 14 days, report the worst one first. Later, you will be able to report the others.

(This is a condensed summary of the questions asked)

1. ID Number
2. Please select the POSITIONS you played in the past 14 days:
3. Please state the date of the first day of your last menstrual period.
4. We are looking at the amount of training sessions you had in the past 14 days.
   According to TIME DURATION, please break down your training sessions in the last 14 days. i.e., for each time period, please select the number of sessions you had (how many).
   (30min-1hour; 1- <2 hours; 2 - <3 hours with options of 1 to 10 sessions for each time frame).
5. How many matches did you play in the past 14 days? (Options: 1 to 5)

Start of block: injuries

6. Did you sustain an INJURY in the past 14 days? An injury can be seen as any physical or structural damage to your body, or physical complaint, affecting your ability to participate in sport

Skip To: End of Block If Did you sustain an injury in the past 14 days? An injury can be seen as any physical or structure... = No

7. Did you have any difficulties participating in training and competition due this injury during the past 14 days? (Full participation without problems; Full participation but with problems; Reduced participation due to injury; Could not participate at all due to injury)

Skip To: End of Block If Did you have any difficulties participating in training and competition due this injury during the... = Full participation without problems

Skip To: Q14 If Did you have any difficulties participating in training and competition due this injury during the... = Could not participate at all due to injury

8. On a scale of 0 to 10, how much did you modify your training or competition due to this injury during the past 14 days?

Skip To: End of Block If On a scale of 0 to 10, how much did you modify your training or competition due to this injury du... = 0

9. On a scale of 0 to 10, to what extent has injury affected your performance in the past 14 days?
10. Was the onset of your injury sudden or gradual? If sudden, please specify the date the injury occurred.
11. Is this the first time you register this injury through this survey?
    (Options: Yes; No, I reported the same injury in the past 4 weeks; No, I reported the same injury more than 4 weeks ago.)
12. Please select the location of your injury:
    (Options: Head/Face; Neck; Shoulder; Upper arm; Elbow; Forearm; Wrist; Hand/Fingers; Chest/rib; Thoracic Spine (Upper back); Lumbar Spine (Lower back); Abdomen; Hip/Groin; Thigh; Knee; Lower Leg; Ankle; Foot/Toes; Other, please specify)
13. Please select the option that best describes your TYPE of injury:
    (Options: Muscle injury, including sprains and tears; Muscle contusion [bruise]; Tendon injury; Brain/Spinal cord injury like concussion; Damaged or trapped nerve; Fracture [Broken bone]; Stress fracture [Overuse fracture due to repetitive impact]; Cartilage injury [Like a meniscus injury]; Joint Sprain [Ligament tear or episode of joint instability]; Skin bruising or wound [like blister, cut, abrasion]; Other, please specify)
14. Did you need medical attention like a physiotherapist or a doctor?
15. When did the injury happen?
    (Options: During Competition; During training; During non-netball related exercise; During activities not involving exercise)
16. Was the injury due to a specific action?
    (Options: Contact with another player from the same team; Contact with another player from the opposite team; Contact with the ball; Contact with a stand-still object like the goal post; Jump/Landing; Sudden change in direction; Recurrence of previous injury; Other, please specify)

17. Please state the number of DAYS you missed training due to the injury:
    (Options: 0 Days; 1-3 Days; 4-7 Days; 8 - 14 Days; 15 - 28 Days [1 month]; 29 - 90 Days [3 months]; 91 - 180 Days [6 months]; More than 180 Days [6 months])
18. Did you experience any other injury in the past 14 days?

End of Block: Injuries

Start of Block: Second injury. A repetition of the same questions as first injury.

Start of Block: Illness

19. Did you contract any illness in the past 14 days?

Skip To: End of Survey If Did you contract any illness in the past 14 days? = No

20. Did you have any difficulties participating in training and competition due this illness during the past 14 days?

(Options: Full participation without problems; Full participation but with problems; Reduced participation due to the illness; Could not participate at all)

Skip To: Q23 If Did you have any difficulties participating in training and competition due this illness during t... = Could not participate at all

21. On a scale of 0 to 10, how much did you modify your training or competition due to this illness during the past 14 days?
22. On a scale of 0 to 10, to what extent has this illness affected your performance in the past 14 days?
23. Please select the date your symptoms started: ____________________
24. Is this the first time you have registered this illness through this monitoring system?
    (Options: Yes; No, I have reported the same illness 4 weeks ago; No, I have reported the same illness more than 4 weeks ago)
25. Please select the major group of symptoms you have experienced during the past 14 days? (You may select more than 1)
    (Options: Upper Respiratory Tract [Flu, Sinusitis, Seasonal allergies, tonsillitis, sore throat, coughing]; Lower Respiratory Tract [Chest congestion, wheezing, shortness of breath, labored breathing, chest pain when breathing or coughing]; Gastrointestinal [Heartburn, nausea, vomiting, abdominal pain or cramps, constipation, diarrhea, blood in stools]; Cardiovascular [Racing heart beats, abnormal or irregular heart beats, chest pain, dizziness, black out or fainting]; Urological [Burning urine, blood in urine, frequent urination, difficulty in passing urine] Gynecological [Loss of normal menstruation, excessively long or heavy menstruation, menstrual cramps, irregular menstruation, vaginal discharge, genital sores, swollen glands in the groin]; Neurological [Headaches, fits or convulsions, pins and needles, muscle weakness, loss of feeling over a specific area]; Psychological [Anxiety, excessive sadness, feeling depressed, excessive restlessness, excessive stress, mood swings, struggling to sleep]; Dermatological [Skin rash, itchy skin lesions, moles that have changed shape or size, skin color change]; Musculoskeletal NOT injury related [Joint pain, swelling, stiffness, warmth or redness, muscle pain and cramps NOT related to exercise]; Dental [Toothache, broken tooth, caries, sensitive teeth, bleeding or sensitive gums, mouth ulcers, painful jaw]; Ears [Ear pain, discharge from ear, new onset decreased hearing, bleeding from ear]; Eyes [Painful, itchy, burning or red eyes, swelling of eye, abnormal tearing, scratchy eye, new onset change in vision, blind spot, abnormal eye movements]; Energy and nutrition [Unexplained under-performance, tiredness and reduced training ability, reduced appetite] Non-specific illness [Nonspecific fever, chills, body pains]; Other, please specify)

26. Do you have a specific diagnosis for your illness?

Skip To: Q28 If Do you have a specific diagnosis for your illness? = No

27. Who made the diagnosis?
    (Options: Doctor; Physiotherapist; Other health care professional; Coach; Self-diagnosed)
28. How was your illness treated?
    (Options: Self-medication; Antibiotics; Other medication prescribed by a doctor; Referral to another health care professional)
29. How many days did you completely miss training or competition due to this illness?
    (Option: 0 Days; 1-3 Days; 4-7 Days; 8 - 14 Days; 15 - 28 Days [1 month]; 29 - 90 Days [3 months] 91 - 180 Days [6 months]; More than 180 Days [6 months])
30. Did you contract any other ILLNESSES in the past 14 days?

End of Block: Illness

Start of Block: Illness 2. A repetition of the same questions as 1st illness.

End of survey
